# Transcatheter Arterial Embolization for Non‐Variceal Upper Gastrointestinal Bleeding: Does the Number of Prior EGDs Matter?

**DOI:** 10.1002/jgh3.70328

**Published:** 2025-12-22

**Authors:** Nurlan Aliyev, Omid Sanaei, Anasua Deb, Aun R. Shah, Kathryn Hutchins, Fedja A. Rochling, Elizabeth R. Lyden, Ishfaq Bhat

**Affiliations:** ^1^ Division of Gastroenterology and Hepatology University of Nebraska Medical Center Omaha Nebraska USA; ^2^ Division of Gastroenterology and Hepatology Creighton University Medical Center Omaha Nebraska USA; ^3^ College of Public Health University of Nebraska Medical Center Omaha Nebraska USA

## Abstract

**Background and Aims:**

Transcatheter arterial embolization (TAE) is an important therapy for non‐variceal upper gastrointestinal bleeding (NVUGIB) when endoscopic intervention fails. The impact of repeated esophagogastroduodenoscopies (EGDs) before TAE on clinical outcomes remains unclear. This study aimed to evaluate the association between the number of EGDs before TAE and outcomes including rebleeding, mortality, and hospitalization.

**Methods:**

We conducted a retrospective cohort study of adults with NVUGIB undergoing TAE at a single tertiary center (2010–2023). Patients were stratified by the number of pre‐TAE EGDs (0, 1, and ≥ 2), embolization strategy (therapeutic, empiric, no embolization), number of embolic agents, and vessel involvement. Primary outcome was 30‐day rebleeding; secondary outcomes included in‐hospital and 30‐day mortality, length of stay, transfusion needs, and cost.

**Results:**

Ninety‐two patients (median age 68; 52.2% male) were included. Thirty‐day rebleeding occurred in 43.3%, and in‐hospital mortality was 14.1%. Patients with ≥ 2 EGDs had the longest hospital stay (14.5 vs. 10 vs. 5.5 days; *p* = 0.0027) without reduced rebleeding or mortality. Rebleeding was highest with no embolization (81.8%) versus empiric (35.8%) or therapeutic (42.3%) embolization (*p* = 0.017). Repeated EGDs were associated with lower embolization rates, while triple embolic agents correlated with highest mortality (66.7%; *p* = 0.0152).

**Conclusions:**

Repeated EGDs before TAE prolonged hospitalization without improving outcomes. Both empiric and therapeutic embolization reduced rebleeding compared with angiography alone. Early referral for TAE after failed endoscopy may optimize NVUGIB management.

## 1 Introduction

Despite declining incidence and mortality over the past three decades, non‐variceal upper gastrointestinal bleeding (NVUGIB) remains a significant medical challenge, affecting approximately 67 per 100 000 individuals annually [1]. It continues to contribute substantially to morbidity, mortality, and healthcare resource utilization [2, 3, 4, 5]. Endoscopic hemostasis is the first‐line approach for NVUGIB after initial stabilization, with success rates exceeding 90% [6]. However, 10%–20% of patients experience rebleeding following initial endoscopy [7, 8], which increases morbidity, mortality, and healthcare costs [9]. In such scenarios, transcatheter arterial embolization (TAE) has emerged as a valuable, minimally invasive treatment option alternative to surgery [10, 11].

Current practice guidelines recommend a second endoscopy before proceeding to TAE in rebleeding cases; however, this is a conditional recommendation based on low‐quality evidence [6, 12]. Notably, no randomized clinical trial to date has compared second endoscopy versus TAE in the setting of recurrent NVUGIB. In real‐world practice, management strategies vary—some clinicians pursue early TAE without repeating endoscopy, while others reserve TAE for persistent bleeding after multiple endoscopic attempts. The impact of the number of endoscopic procedures prior to angiography on clinical outcomes remains poorly understood.

TAE is typically reserved for patients in whom endoscopic therapy has failed or is not feasible due to anatomical limitations or coagulopathy [13, 14]. Technological advances in microcatheters and embolic materials over the past two decades have significantly improved the safety and efficacy of TAE, with technical success rates of 80%–100% and clinical success rates of 70%–90% [15, 16, 17, 18, 19, 20]. When active contrast extravasation is identified on angiography, therapeutic (targeted) TAE is performed. In the absence of visible bleeding, empiric embolization may be undertaken at the discretion of the interventional radiologist. This approach is used when angiography does not reveal active hemorrhage, but clinical, endoscopic, or radiologic evidence strongly suggests a bleeding source. In contrast, prophylactic embolization, which may be considered for high‐risk lesions following successful endoscopic hemostasis, is outside the scope of this analysis.

This study specifically focuses on patients with concern for active NVUGIB who underwent either therapeutic or empiric embolization.

This study aims to assess:

1. The impact of pre‐angiographic EGD(s) on clinical outcomes of TAE in NVUGIB.
2. The relationship between pre‐angiographic EGD(s) and embolization strategies (therapeutic, empiric, or no embolization).
3. The effect of embolization strategies on clinical outcomes.
4. The influence of embolic agents (single, double, or triple) and embolized vessels on clinical outcomes.

## 2 Methods

### 2.1 Study Design

This was a single‐center retrospective study of patients diagnosed with NVUGIB who underwent transcatheter arterial embolization (TAE) from January 2010 to August 2023 at the University of Nebraska Medical Center.

### 2.2 Reporting Guidelines and IRB

This study follows the Strengthening the Reporting of Observational Studies in Epidemiology guidelines for the retrospective cohort study. The internal review board at the University of Nebraska Medical Center has approved this study (IRB protocol # 0180‐23‐EP).

### 2.3 Data Source and Data Collection

Data was extracted from the UNMC electronic medical records. We collected the following data: patient demographics (age at the time of TAE, gender, race, and ethnicity), comorbidities (end‐stage renal disease, chronic kidney disease, cirrhosis, cancer, hypertension, and diabetes mellitus), level of care, location of care, admission and discharge date and time, laboratory results, vital signs, medication administration, type and amount of blood products transfused, TAE procedures, hospital LOS, ICU LOS, and mortality during hospitalization and within 30 days of discharge. A chart review of individuals estimates 30‐day rebleeding.

### 2.4 Participants and Setting

We included all hospitalized patients age 19 and above with a diagnosis of acute NVUGIB in the study period. In this study, NVUGIB was defined as acute upper gastrointestinal bleeding originating proximally to the ligament of Treitz, excluding variceal sources. NVUGIB was identified using International Classification of Diseases, 10th Revision (ICD‐10) codes. TAE procedures were determined based on Current Procedural Terminology (CPT) codes and associated surgical identifiers (surgery IDs). A detailed list of the ICD‐10 codes, CPT codes, and surgery IDs is provided in Table S1.

We excluded patients who had TAE for conditions other than NVUGIB. For instance, patients who underwent embolization for tumors, bleeding arteriovenous fistulas, arteriovenous malformations, aneurysms, and pseudoaneurysms outside the GI tract were excluded. One procedure was unsuccessful due to celiac artery stenosis and was excluded from the analysis as well.

**FIGURE 1:**
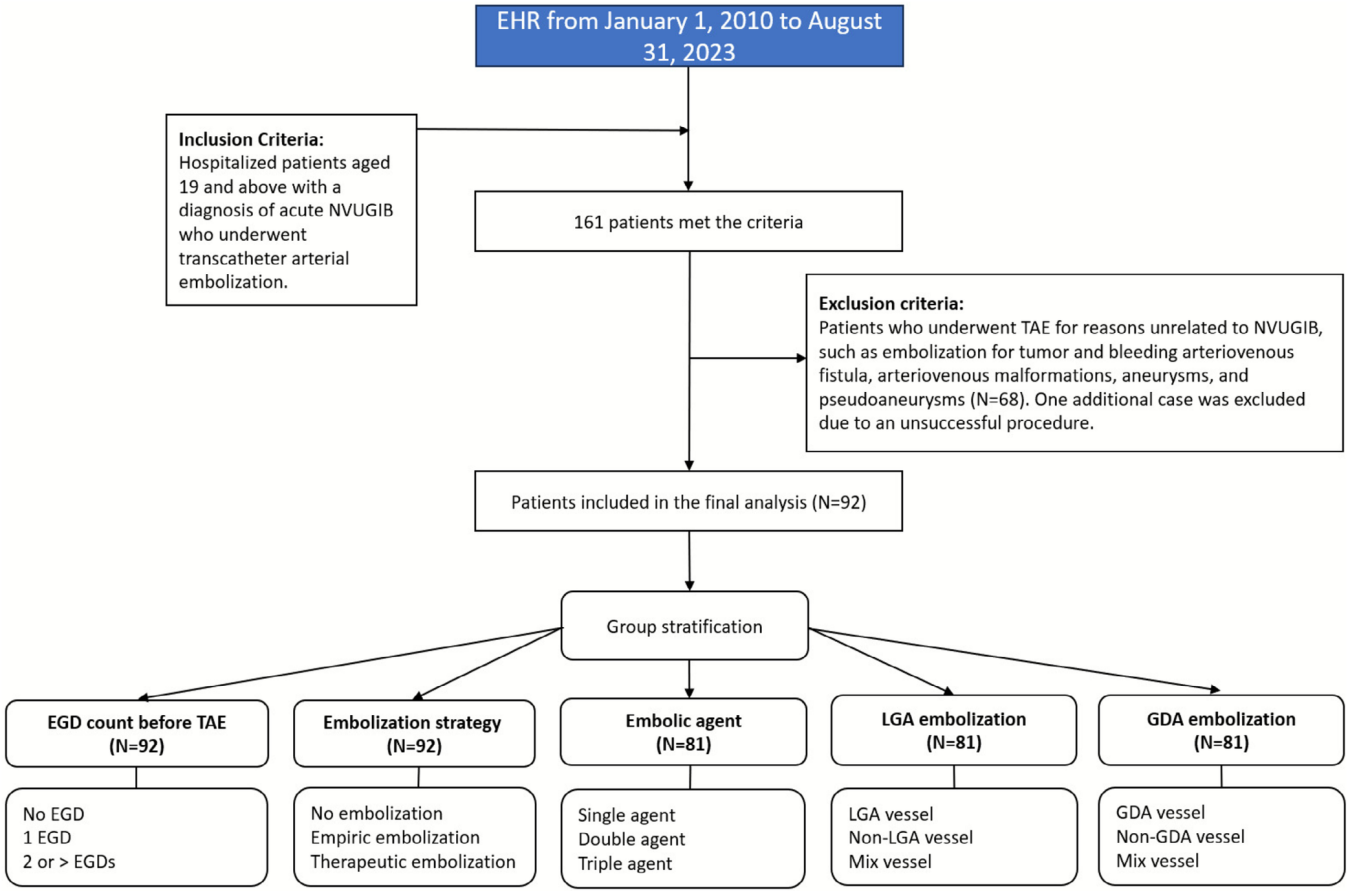
Selection flowchart.

Each TAE procedure report was reviewed, and data was then stratified into the following subgroups: (1) Number of EGDs performed before TAE (no EGD, 1 EGD, and 2 or more EGDs); (2) embolization approach (no embolization, prophylactic embolization, and therapeutic embolization); (3) Number of embolic agents used (single, double, and triple agents); (4) Vessel intervened (LGA and non‐LGA vessel) and (GDA and non‐GDA vessel) (Figure 1).

### 2.5 Outcomes

The primary outcome was a 30‐day rebleeding rate following TAE. The 30‐day rebleeding rate was determined via individual chart review and uniform application of the following criteria:

- Recurrence of bleeding within 30 days of TAE.
- Ongoing melena, hematemesis, hematochezia, acute blood loss anemia, and iron deficiency anemia.
- Hemoglobin drops of more than 2 g/dL or at least a unit of packed red blood cells transfusion were given to maintain hemoglobin.
- Imaging findings of active bleeding.
- High‐risk stigmata of bleeding on endoscopic evaluation.
- No alternative sources of bleeding were clinically suspected other than NVUGIB.
- No alternative sources of bleeding were found upon further workup.

When clinically feasible, rebleeding episodes were further evaluated with repeat endoscopy; however, confirmatory endoscopy was not universally performed in all cases.

Secondary outcome measures included death from any cause during hospitalization and within 30 days of hospital discharge, total hospital and ICU length of stay (LOS), packed red blood cells (PRBC) transfusion requirement, and total cost of admission. The total cost of admission is calculated with the total sum of charges associated with the admission encounter.

### 2.6 Statistical Analysis

Descriptive statistics (means, standard deviations, medians, counts, and percentages) were used to summarize demographic characteristics. Patient characteristics and outcomes were compared between the study groups using Fisher's test for categorical variables. For continuous variables, the Wilcoxon rank sum test was used for the dichotomous study groups, and the Kruskal‐Wallis test was used for the study groups with > 2 levels. Pairwise comparison was performed using Fisher's Exact Test, with *p* values adjusted using the Bonferroni method. Analyses were done using SAS, Version 9.4. *p* < 0.05 was considered statistically significant.

## 3 Results

We first described the study cohort by examining the baseline characteristics, including demographics and comorbidities. Outcomes related to in‐hospital and 30‐day mortality, rebleeding, LOS, red blood cell transfusion, and cost of care were then analyzed. Subgroup analyses were also performed, as described below.

### 3.1 Description of Study Cohort

#### 3.1.1 Baseline Characteristics

A total of 161 patients underwent TAE, of whom 68 were excluded due to embolization for tumor, bleeding arteriovenous fistula, arteriovenous malformations, aneurysms, or pseudoaneurysms. One additional case was excluded due to an unsuccessful procedure secondary to celiac artery stenosis. CA was successful in 92 out of 93 patients (98.9%).

The final cohort included 92 patients, with a median age of 68 years; 47.8% were female and 52.2% male. The majority were White/Caucasian (78%). Common comorbidities included hypertension (64.1%), diabetes mellitus (32.6%), cancer (29.3%), chronic kidney disease (19.6%), liver cirrhosis (5.4%), end‐stage renal disease (4.3%) (Table 1).

**TABLE 1:** Descriptive statistics.

Characteristics	Total (*N* = 92)	Outcomes measures	Total (*N* = 92)
Age		Death during hospitalization, *n* (%)	13 (14.1%)
*N*	92		
Mean (SD)	64.72 (14.61)	Death within 30‐day of discharge, *n* (%)	6 (6.5%)
Median (Range)	68.00 (19.0, 93.0)		
IQR	57.50, 74.00	30‐day rebleeding, *n* (%)	39 (43.3%)
Gender, *n* (%)		Hospital LOS days	
Female	44 (47.8%)	Mean (SD)	18.37 (20.71)
Male	48 (52.2%)	Median (Range)	12.00 (1.0, 113.0)
		IQR	6.00, 23.00
Race, *n* (%)			
American Indian or Alaska Native	3 (3.3%)	ICU LOS days	
Asian	3 (3.3%)	Mean (SD)	6.86 (11.45)
Black or African American	10 (11.0%)	Median (Range)	3.00 (0.0, 60.0)
Other	4 (4.4%)	IQR	1.00, 7.00
White or Caucasian	71 (78.0%)		
		Total admission cost ($)	
Ethnicity, *n* (%)		Mean (SD)	41188.92 (32196.03)
Hispanic or Latino	3 (3.3%)	Median (Range)	34805.35 (1000.0, 181063.0)
Not Hispanic or Latino	89 (96.7%)	IQR	19429.00, 49626.31
Comorbidities		PRBC transfusion (mL)	
ESRD, *n* (%)	4 (4.3%)	Mean (SD)	2037.08 (2155.09)
CKD, *n* (%)	18 (19.6%)	Median (Range)	1050.00 (286.0, 7700.0)
Cirrhosis, *n* (%)	5 (5.4%)	IQR	563.00, 2853.00
Cancer, *n* (%)	27 (29.3%)		
DM, *n* (%)	30 (32.6%)		
HTN, *n* (%)	59 (64.1%)		
EGD before TAE, *n* (%)			
No EGD	12 (13.0%)		
1 EGD	50 (54.3%)		
≥ 2 EGDs	30 (32.6%)		
Embolization approach, *n* (%)			
No embolization	11 (12.0%)		
Empiric embolization	54 (58.7%)		
Therapeutic embolization	27 (29.3%)		
Embolic agent, *n* (%)			
Single agent	70 (86.4%)		
Double agent	8 (9.9%)		
Triple agent	3 (3.7%)		
LGA embolization, *n* (%)			
LGA	9 (11.1%)		
Non‐LGA	68 (84.0%)		
Mix	4 (4.9%)		
GDA embolization, *n* (%)			
GDA	40 (49.4%)		
Non‐GDA	35 (43.2%)		
Mix	6 (7.4%)		

#### 3.1.2 Outcomes

In‐hospital mortality occurred in 14.1% of patients, with an additional 6.5% dying within 30 days post‐discharge. Rebleeding within 30 days occurred in 43.3% of patients. The median hospital LOS was 12 days (range: 1–113), and median ICU LOS was 3 days (range: 0–60). The median hospital cost was $34 805 (range: $1000 to $181063).

#### 3.1.3 Interventions

Prior to TAE, 13% (*n* = 12) of patients had no EGD, 54.3% (*n* = 50) had one EGD, and 32.6% (*n* = 30) had ≥ 2 EGDs. In terms of embolization strategy, 12% (*n* = 11) underwent angiography without embolization, 58.7% (*n* = 54) had empiric embolization, and 29.3% (*n* = 27) underwent therapeutic embolization. Most patients (86.4%) received a single embolic agent, 9.9% received two agents, and 3.7% received three agents.

### 3.2 Impact of EGD(s) on Clinical Outcomes

Patients were stratified by the number of pre‐TAE EGDs (Table 2): 12 had no EGD, 50 had one, and 30 had ≥ 2. Baseline characteristics were comparable across groups.

- **30‐day rebleeding** trend was highest in ≥ 2 EGD group (56.7%), followed by the 1 EGD (38.8%) and no EGD (27.3%) groups (*p* = 0.1639).
- **In‐hospital mortality** trended higher in the no‐EGD group (33.3%) compared to 1 EGD (8%) and ≥ 2 EGD (16.7%) (*p* = 0.0618).
- **Median hospital LOS** was significantly higher in ≥ 2 EGD groups (14.5 days) versus 1 EGD (10 days) and no EGD groups (5.5 days) (*p* = 0.0027).
- No significant differences were observed in other clinical outcomes.

**TABLE 2:** EGD(s) before TAE.

Baseline characteristics	No EGD (*N* = 12)	1 EGD (*N* = 50)	≥ 2 EGDs (*N* = 30)	*p*
Age				0.3684^a^
*N*	12	50	30	
Mean (SD)	69.17 (11.52)	62.84 (15.72)	66.07 (13.63)	
Median (Range)	69.00 (46.0, 84.0)	67.00 (26.0, 93.0)	66.50 (19.0, 86.0)	
Gender, *n* (%)				0.5145^b^
Female	7 (58.3%)	25 (50.0%)	12 (40.0%)	
Male	5 (41.7%)	25 (50.0%)	18 (60.0%)	
Ethnicity, *n* (%)				1.000^b^
Hispanic or Latino	0 (0.0%)	2 (4.0%)	1 (3.3%)	
Not Hispanic or Latino	12 (100.0%)	48 (96.0%)	29 (96.7%)	
Race, *n* (%)				0.8888^b^
American Indian or Alaska Native	0 (0.0%)	3 (6.0%)	0 (0.0%)	
Asian	0 (0.0%)	2 (4.0%)	1 (3.3%)	
Black or African American	1 (9.1%)	4 (8.0%)	5 (16.7%)	
Other	0 (0.0%)	3 (6.0%)	1 (3.3%)	
White or Caucasian	10 (90.9%)	38 (76.0%)	23 (76.7%)	
ESRD, *n* (%)	0 (0.0%)	1 (2.0%)	3 (10.0%)	0.1810^b^
CKD, *n* (%)	1 (8.3%)	10 (20.0%)	7 (23.3%)	0.6584^b^
Cirrhosis, *n* (%)	1 (8.3%)	2 (4.0%)	2 (6.7%)	0.5427^b^
Cancer, *n* (%)	4 (33.3%)	15 (30.0%)	8 (26.7%)	0.9009^b^
DM, *n* (%)	5 (41.7%)	11 (22.0%)	14 (46.7%)	0.0569^b^
HTN, *n* (%)	6 (50.0%)	31 (62.0%)	22 (73.3%)	0.3293^b^
Study outcomes				
30‐day rebleeding, *n* (%)	3 (27.3%)	19 (38.8%)	17 (56.7%)	0.1639^b^
Death within 30 days of discharge, *n* (%)	0 (0.0%)	5 (10.0%)	1 (3.3%)	0.4882^b^
Death during hospitalization, *n* (%)	4 (33.3%)	4 (8.0%)	5 (16.7%)	0.0618^b^
Hospital LOS days				0.0027^a^
*N*	12	50	30	
Mean (SD)	8.08 (9.02)	18.76 (23.17)	21.83 (18.73)	
Median (Range)	5.50 (1.0, 33.0)	10.00 (1.0, 113.0)	14.50 (4.0, 75.0)	
ICU LOS days				0.4172^a^
*N*	12	50	30	
Mean (SD)	3.17 (4.09)	6.46 (10.85)	9.00 (13.98)	
Median (Range)	2.00 (0.0, 15.0)	3.00 (0.0, 59.0)	4.00 (0.0, 60.0)	
Total admission cost ($)				0.7404^a^
*N*	11	48	29	
Mean (SD)	37730.54 (27819.57)	37941.02 (24780.02)	47876.57 (42948.60)	
Median (Range)	28263.98 (4281.0, 79550.6)	35095.30 (1000.0, 120969.3)	33835.50 (1005.0, 181063.0)	
PRBC transfusion (mL)				0.9173^a^
*N*	3	13	8	
Mean (SD)	933.33 (202.07)	2222.23 (2201.17)	2150.13 (2518.37)	
Median (Range)	1050.00 (700.0, 1050.0)	1650.00 (286.0, 7700.0)	875.00 (350.0, 7350.0)	

^a^ Kruskal–Wallis *p* value.

^b^ Fisher exact *p* value.

### 3.3 Impact of Embolization Strategy on Clinical Outcomes

Patients were categorized into three groups based on embolization strategy (Table 3): no embolization (*n* = 11), empiric embolization (*n* = 54), and therapeutic embolization (*n* = 27). Baseline characteristics were similar, except for a higher prevalence of end‐stage renal disease in the no‐embolization group.

- 30‐day rebleeding was significantly higher in the no‐embolization group (81.8%) compared to empiric (35.8%) and therapeutic embolization (42.3%) groups (*p* = 0.017).
- In‐hospital mortality was 27.3% in the non‐embolization group, 9.3% in empiric, and 18.5% in therapeutic groups (*p* = 0.179).
- Median hospital LOS was longest in the non‐embolization group (23 days), compared to 9.5 and 10 days in the empiric and therapeutic embolization groups, respectively (*p* = 0.017).
- Other outcomes, including post‐discharge mortality, ICU LOS, cost, and transfusion requirements, were comparable across groups.

**TABLE 3:** Embolization strategies.

Baseline characteristics	No embolization (*N* = 11)	Empiric embolization (*N* = 54)	Therapeutic embolization (*N* = 27)	*p*
Age				0.671^a^
*N*	11	54	27	
Mean (SD)	68.18 (11.58)	63.28 (16.13)	66.19 (12.41)	
Median (Range)	68.00 (55.0, 84.0)	67.00 (19.0, 93.0)	68.00 (31.0, 86.0)	
Gender, *n* (%)				1.000^b^
Female	5 (45.5%)	26 (48.1%)	13 (48.1%)	
Male	6 (54.5%)	28 (51.9%)	14 (51.9%)	
Ethnicity, *n* (%)				1.000^b^
Hispanic or Latino	0 (0.0%)	2 (3.7%)	1 (3.7%)	
Not Hispanic or Latino	11 (100.0%)	52 (96.3%)	26 (96.3%)	
Race, *n* (%)				0.092^b^
American Indian or Alaska Native	0 (0.0%)	3 (5.6%)	0 (0.0%)	
Asian	0 (0.0%)	3 (5.6%)	0 (0.0%)	
Black or African American	4 (36.4%)	2 (3.7%)	4 (15.4%)	
Other	0 (0.0%)	3 (5.6%)	1 (3.8%)	
White or Caucasian	7 (63.6%)	43 (79.6%)	21 (80.8%)	
ESRD, *n* (%)	3 (27.3%)	0 (0.0%)	1 (3.7%)	0.002^b^
CKD, *n* (%)	1 (9.1%)	11 (20.4%)	6 (22.2%)	0.751^b^
Cirrhosis, *n* (%)	0 (0.0%)	5 (9.3%)	0 (0.0%)	0.231^b^
Cancer, *n* (%)	4 (36.4%)	18 (33.3%)	5 (18.5%)	0.361^b^
DM, *n* (%)	6 (54.5%)	16 (29.6%)	8 (29.6%)	0.289^b^
HTN, *n* (%)	8 (72.7%)	37 (68.5%)	14 (51.9%)	0.276^b^
Study outcomes				
30‐day rebleeding, *n* (%)	9 (81.8%)	19 (35.8%)	11 (42.3%)	0.017^b^
Death within 30 days of discharge, *n* (%)	0 (0.0%)	5 (9.3%)	1 (3.7%)	0.578^b^
Death during hospitalization, *n* (%)	3 (27.3%)	5 (9.3%)	5 (18.5%)	0.179^b^
Hospital LOS days				0.017^a^
*N*	11	54	27	
Mean (SD)	28.82 (19.74)	18.22 (23.38)	14.41 (13.19)	
Median (Range)	23.00 (12.0, 75.0)	9.50 (1.0, 113.0)	10.00 (1.0, 55.0)	
ICU LOS days				0.122^a^
*N*	11	54	27	
Mean (SD)	10.82 (17.87)	6.09 (11.32)	6.78 (8.20)	
Median (Range)	5.00 (0.0, 60.0)	2.50 (0.0, 59.0)	4.00 (0.0, 37.0)	
Total admission cost ($)				0.805^a^
*N*	11	51	26	
Mean (SD)	44749.06 (48463.26)	39704.13 (30247.11)	42595.19 (28739.03)	
Median (Range)	30146.40 (11236.1, 181063.0)	34702.20 (1005.0, 168643.1)	37083.26 (1000.0, 119366.7)	
PRBC transfusion (mL)				0.942^a^
*N*	3	10	11	
Mean (SD)	2800.00 (3944.30)	2376.00 (2554.68)	1520.91 (1055.62)	
Median (Range)	700.00 (350.0, 7350.0)	1082.00 (350.0, 7700.0)	1050.00 (286.0, 3150.0)	

^a^ Kruskal–Wallis *p* value.

^b^ Fisher exact *p* value.

### 3.4 Impact of EGD(s) on Embolization Strategy

Further analysis was conducted to assess the impact of pre‐angiogram EGD(s) on embolization strategies (no embolization, empiric, and therapeutic embolization) (Table 4). Pairwise comparisons showed significant differences in embolization strategy between patients who had 0 EGD versus ≥ 2 EGDs (*p* = 0.0073) and between patients with 1 EGD versus ≥ 2 EGDs (*p* = 0.0023). Most patients who did not receive embolization (81.8%) had ≥ 2 EGD before CA, while most patients who received empiric (63%) and therapeutic (54.3%) embolization had 1 EGD before CA (*p* < 0.0001).

**TABLE 4:** Pairwise comparisons.

Association of EGDs with embolization strategies (column percentage)					
	No embolization (*N* = 11)	Empiric embolization (*N* = 54)	Therapeutic embolization (*N* = 27)	Total (*N* = 92)	*p*
Number of EGDs, *n* (%)					< 0.0001^a^
0 EGD	1 (9.1%)	3 (5.6%)	8 (29.6%)	12 (13.0%)	
1 EGD	1 (9.1%)	34 (63.0%)	15 (55.6%)	50 (54.3%)	
≥ 2 EGDs	9 (81.8%)	17 (31.5%)	4 (14.8%)	30 (32.6%)	

Association of EGDs with embolization strategies (row percentage)					
	No embolization (*N* = 11)	Empiric embolization (N = 54)	Therapeutic embolization (*N* = 27)	Total (*N* = 92)	*p*
Number of EGDs, *n* (%)					<.0001^a^
0 EGD	1 (8.3%)	3 (25.0%)	8 (66.7%)	12 (13.0%)	
1 EGD	1 (2.0%)	34 (68.0%)	15 (30.0%)	50 (54.3%)	
≥ 2 EGDs	9 (30.0%)	17 (56.7%)	4 (13.3%)	30 (32.6%)	

Pairwise comparisons between EGDs after adjustment	
Pair compared	*p*
0 EGD vs. 1 EGD	0.064
0 EGD vs.≥ 2 EGDs	0.0073
1 EGD vs. ≥ 2 EGDs	0.0023

^a^ Fisher Exact *p* value.

In the empiric embolization group, no EGD group had higher in‐hospital mortality than 1 EGD and ≥ 2 EGDs (66.7%, 2.9%, and 11.8%, *p* = 0.010, respectively). In contrast, no EGD had a shorter hospital LOS, which was attributed to high in‐hospital mortality. Median hospital LOS was lower in the 1 EGD group than ≥ 2 EGD (8.5 vs. 14, *p* = 0.018). In no embolization and therapeutic embolization groups, the number of scopes before the embolization did not significantly impact the outcome (Table S2).

### 3.5 Impact of Embolic Agents on Clinical Outcomes

Among 81 patients who received embolization: 70 (86.4%) received a single embolic agent, 8 (9.9%) received two, and 3 (3.7%) received three agents. While baseline characteristics were largely similar, hypertension and cancer were more common in those receiving multiple agents. In‐hospital mortality was highest in patients receiving triple agents (66.7%) compared to double (25%) and single‐agent groups (8.6%) (*p* = 0.0152).

### 3.6 Impact of LGA Embolization on Clinical Outcomes

Of the 81 embolized patients: 9 (11.1%) underwent LGA embolization, 68 (84%) had non‐LGA embolization, and 4 (4.9%) had mixed LGA and non‐LGA embolization. Baseline characteristics and overall outcomes were similar. However, patients with mixed‐vessel embolization had significantly higher pRBC transfusion volumes compared to the non‐LGA group (1149 mL vs. 6475 mL, *p* = 0.0226). The data is available on request.

### 3.7 Impact of GDA Vessel Embolization on Clinical Outcomes

Among the same 81 patients: 40 (49.4%) had GDA embolization, 35 (43.2%) had non‐GDA embolization, and 6 (7.4%) underwent mixed‐vessel embolization. Baseline characteristics were generally similar, except that liver cirrhosis was more prevalent in mixed (16.7%) and non‐GDA (11.4%) groups compared to the GDA group (0%) (*p* = 0.0241). Notably, median ICU LOS was highest in the mixed group (10.5 days) compared to GDA (3 days) and non‐GDA (2 days) groups (*p* = 0.0458). The data is available on request.

## 4 Discussion

This retrospective analysis evaluated the clinical outcomes and procedural characteristics of patients undergoing TAE for NVUGIB. Our findings highlight a significant correlation between the number of endoscopic evaluations, embolization strategies, and clinical outcomes.

The study cohort predominantly included elderly patients with substantial comorbidities—such as hypertension, diabetes, and malignancy—consistent with prior studies describing high‐risk NVUGIB populations [21]. Although catheter angiography demonstrated a high technical success rate (98.9%), only 88% of patients ultimately underwent embolization. Overall, the cohort exhibited a high in‐hospital mortality rate (14.1%) and a 30‐day rebleeding rate (43.3%), underscoring the clinical challenges in managing this critically ill group. These adverse outcomes may partly be attributed to the relatively low embolization rate and the high burden of comorbidities.

Among those who underwent embolization, the 30‐day rebleeding rate was 37%. In comparison, a systematic review and meta‐analysis by Matsumoto et al. involving 989 cases reported a pooled 30‐day rebleeding rate of 25% (95% CI: 19.5%–31.3%) and a higher 30‐day mortality rate of 20.7% (95% CI: 13.2%–31.1%) [22]. These discrepancies likely reflect differences in patient demographics, comorbidity profiles, and interventional expertise.

Endoscopic evaluation prior to TAE plays a crucial role in determining the bleeding source, delineating anatomy, and placing endoscopic clips to mark sites for targeted angiography [23]. This is especially valuable when extravasation is not visible on angiography, thereby improving the likelihood of successful empiric embolization [24]. However, the clinical utility of repeated endoscopy before proceeding to TAE remains unclear, and no randomized trials have directly compared second‐look EGD with early embolization.

To our knowledge, this is the first study to explore the impact of repeated EGD versus early TAE following initial endoscopic failure in NVUGIB. Our findings suggest that undergoing ≥ 2 EGDs prior to embolization is associated with prolonged hospitalization without a corresponding reduction in rebleeding or mortality. These results imply that repeated EGDs may reflect ongoing diagnostic uncertainty or persistent hemorrhage, but they do not necessarily lead to improved clinical outcomes. Previous studies have similarly proposed that early embolization may be more effective than repeated endoscopic interventions in high‐risk bleeding scenarios [25, 26].

Empiric TAE remains controversial due to limited high‐quality evidence. While conservative management may be effective in some patients with negative angiography—reportedly up to 80% avoid rebleeding [27]—several studies have demonstrated improved outcomes with empiric embolization compared to no embolization [28, 29]. A recent meta‐analysis concluded that empiric and targeted embolization offers comparable outcomes [30].

Our study supports these findings: patients undergoing empiric or therapeutic embolization had significantly lower rebleeding rates and shorter hospital stays than those undergoing angiography alone. Notably, patients who did not undergo embolization experienced the highest 30‐day rebleeding rate (81.8%), emphasizing the clinical importance of performing embolization when feasible.

The no‐embolization group had a higher prevalence of end‐stage renal disease, which may have influenced clinical decision‐making and outcomes. In these cases, embolization was deferred because angiographic findings were negative at the time of the procedure. Notably, the markedly higher rebleeding rate observed in this group suggests that the absence of angiographic extravasation does not reliably exclude clinically significant hemorrhage.

Patients who underwent empiric embolization without preceding endoscopy likely represent a high‐risk subgroup in whom endoscopy was not feasible or was deferred due to hemodynamic instability or the need for urgent hemorrhage control. This pragmatic approach underscores the role of empiric embolization as a salvage strategy in critically ill patients when conventional diagnostic pathways are not safely achievable.

We also examined how the number of pre‐angiographic EGDs influenced the embolization strategy. This is the first report to demonstrate that patients who underwent multiple EGDs were less likely to receive embolization, despite undergoing angiography. This suggests that delays in referral or overreliance on repeated endoscopic evaluation may result in missed opportunities for definitive intervention. Early recognition of endoscopic failure and prompt referral to interventional radiology should be emphasized in clinical practice.

Our analysis also evaluated the relationship between the number of embolic agents and clinical outcomes. Although most patients received single‐agent embolization, those who received multiple agents—particularly triple‐agent embolization—had higher in‐hospital mortality (66.7%). This likely reflects greater bleeding severity or procedural complexity rather than the embolic strategy itself. Nonetheless, aggressive embolization should be approached cautiously in patients with limited physiological reserve.

Finally, vessel‐specific analyses, a previous study by Joseph et al. has shown that LGA embolization is associated with higher rebleeding and mortality rates compared to non‐LGA embolization [16]. In our analysis, we identified a third group of patients who underwent embolization of both LGA and non‐LGA vessels (referred to as the mixed group), in addition to those who underwent isolated LGA and non‐LGA embolization. We did not observe significant differences in rebleeding or mortality rates among the three groups (LGA vs. non‐LGA vs. mixed). However, patients in the mixed embolization group required significantly more PRBC transfusions, which may reflect more severe or persistent hemorrhage in this subgroup.

GDA is the most commonly embolized vessel in NVUGIB. In our analysis, the mixed‐GDA embolization group had a longer ICU LOS than the GDA and non‐GDA groups. Notably, mixed‐GDA patients had a higher prevalence of liver cirrhosis, which may further complicate management due to underlying coagulopathy and hemodynamic instability [31].

This study has several limitations. First, its retrospective, single‐center design may limit the generalizability of the findings. Although the sample size was adequate for subgroup analysis, it may have limited the statistical power for certain comparisons. Additionally, the lack of detailed data on coagulopathy, thrombocytopenia, anticoagulant, antiplatelet use, non‐steroidal anti‐inflammatory drug (NSAID) exposure, as well as characteristics of NVUGIB etiologies, restricts interpretation of contributing risk factors. The absence of long‐term follow‐up beyond 30 days also precludes evaluation of delayed complications or reintervention rates. Importantly, the time interval between index endoscopy and TAE was not systematically captured, limiting our ability to evaluate the impact of earlier versus delayed embolization on clinical outcomes. Finally, unmeasured variables—such as timing of embolization, the extent of resuscitation, and procedural expertise—may have influenced the outcomes. These limitations reflect real‐world practice in critically ill patients and should be considered when interpreting outcomes.

## 5 Conclusion

In patients with NVUGIB undergoing angiography, both empiric and therapeutic embolization were associated with improved outcomes compared to angiography alone. Repeated endoscopic evaluations prior to embolization were not associated with reductions in rebleeding or mortality but were associated with prolonged hospital length of stay. These findings suggest that prompt consideration of TAE following failed endoscopic therapy may help avoid delays in definitive treatment. Prospective multicenter studies incorporating precise procedural timing are needed to define the optimal interval between endoscopy and embolization and to validate these observations.

## Funding

This study did not receive any specific funding from public, commercial, or not‐for‐profit funding agencies.

## Conflicts of Interest

The authors declare no conflicts of interest.

## Supporting information


**Table S1:** Selection criteria and codes.
**Table S2:** Outcomes by EGD(s) and Embolization Strategies.

## Data Availability

The data that support the findings of this study are available on request from the corresponding author. The data are not publicly available due to privacy or ethical restrictions.
