# Supplementary material for: Transcatheter Arterial Embolization for Non‐Variceal Upper Gastrointestinal Bleeding: Does the Number of Prior EGDs Matter?
Source: JGH Open. 2025 Dec 22;9(12):e70328. doi: 10.1002/jgh3.70328 (PMC12720415; doi:10.1002/jgh3.70328)
Supplement: Supplementary file 1 — Table S1: Selection criteria and codes. Table S2: Outcomes by EGD(s) and Embolization Strategies. [file JGH3-9-e70328-s001.docx]

**Supplementary Table 1. Selection Criteria and Codes**

| **TAE Procedure Codes (CPT)** | 37241, 37242, 37243, 37244 |
| --- | --- |
| **TAE Procedure Codes (Surgery ID)** | 2568, 2569, 3154, 740, 643 |
|  |  |
| **ICD-10 Codes for NVUGIB** |  |
| K20.81 | Other esophagitis with bleeding |
| K20.91 | Esophagitis, unspecified with bleeding |
| K21.01 | GERD with esophagitis, with bleeding |
| K22.3 | Perforation of esophagus |
| K22.6 | Gastro-esophageal laceration-hemorrhage syndrome |
| K22.11 | Ulcer of esophagus with bleeding |
| K25.0 | Acute gastric ulcer with hemorrhage |
| K25.2 | Acute gastric ulcer with both hemorrhage and perforation |
| K25.4 | Chronic or unspecified gastric ulcer with hemorrhage |
| K25.6 | Chronic or unspecified gastric ulcer with both hemorrhage and perforation |
| K26.0 | Acute duodenal ulcer with hemorrhage |
| K26.2 | Acute duodenal ulcer with both hemorrhage and perforation |
| K26.4 | Chronic or unspecified duodenal ulcer with hemorrhage |
| K26.6 | Chronic or unspecified duodenal ulcer with both hemorrhage and perforation |
| K27.0 | Acute peptic ulcer, site unspecified, with hemorrhage |
| K27.2 | Acute peptic ulcer, site unspecified, with both hemorrhage and perforation |
| K27.4 | Chronic or unspecified peptic ulcer, site unspecified, with hemorrhage |
| K27.6 | Chronic or unspecified peptic ulcer, site unspecified, with both hemorrhage and perforation |
| K28.0 | Acute gastrojejunal ulcer with hemorrhage |
| K28.2 | Acute gastrojejunal ulcer with both hemorrhage and perforation |
| K28.4 | Chronic or unspecified gastrojejunal ulcer with hemorrhage |
| K28.6 | Chronic or unspecified gastrojejunal ulcer with both hemorrhage and perforation |
| K29.01 | Acute gastritis with bleeding |
| K29.21 | Alcoholic gastritis with bleeding |
| K29.31 | Chronic superficial gastritis with bleeding |
| K29.41 | Chronic atrophic gastritis with bleeding |
| K29.51 | Unspecified chronic gastritis with bleeding |
| K29.61 | Other gastritis with bleeding |
| K29.71 | Gastritis, unspecified, with bleeding |
| K29.81 | Duodenitis with bleeding |
| K29.91 | Gastroduodenitis, unspecified, with bleeding |
| K31.811 | Angiodysplasia of stomach and duodenum with bleeding |
| K31.82 | Dieulafoy lesion (hemorrhagic) of stomach and duodenum |
| K92.0 | Hematemesis |
| K92.1 | Melena |
| K92.2 | Gastrointestinal hemorrhage, unspecified |

**Supplementary Table 2. Outcomes by EGD(s) and Embolization Strategies**

|  | | ***EGD(s)*** | | |  |
| --- | --- | --- | --- | --- | --- |
| ***Embolization Strategies*** | ***Outcomes*** | ***0 EGD*** | ***1 EGD*** | ***>=2 EGDs*** | ***P-value*** |
| **Angiogram only** |  | **(N=1)** | **(N=1)** | **(N=9)** |  |
|  | 30-day rebleeding, n (%) | 1 (100.0%) | 0 (0.0%) | 8 (88.9%) | 0.345^1^ |
|  | Death within 30-day of discharge, n (%) | 0 (0.0%) | 0 (0.0%) | 0 (0.0%) |  |
|  | Death during hospitalization, n (%) | 0 (0.0%) | 0 (0.0%) | 3 (33.3%) | 1.000^1^ |
|  | Hospital LOS days |  |  |  | 0.254^2^ |
|  | N | 1 | 1 | 9 |  |
|  | Median | 33.0 | 12.0 | 23.0 |  |
|  | Range | 33.0, 33.0 | 12.0, 12.0 | 13.0, 75.0 |  |
|  | ICU LOS days |  |  |  | 0.633^2^ |
|  | N | 1 | 1 | 9 |  |
|  | Median | 5.0 | 1.0 | 6.0 |  |
|  | Range | 5.0, 5.0 | 1.0, 1.0 | 0.0, 60.0 |  |
|  | Total admission cost ($) |  |  |  | 0.336^2^ |
|  | N | 1 | 1 | 9 |  |
|  | Median | 19347.0 | 14353.8 | 40300.2 |  |
|  | Range | 19347.0, 19347.0 | 14353.8, 14353.8 | 11236.1, 181063.0 |  |
|  | PRBC transfusion (mL) |  |  |  | 0.221^2^ |
|  | N |  | 1 | 2 |  |
|  | Median |  | 350.0 | 4025.0 |  |
|  | Range |  | 350.0, 350.0 | 700.0, 7350.0 |  |
| **Empiric embolization** |  | **(N=3)** | **(N=34)** | **(N=17)** |  |
|  | 30-day rebleeding, n (%) | 1 (50.0%) | 11 (32.4%) | 7 (41.2%) | 0.693^1^ |
|  | Death within 30-day of discharge, n (%) | 0 (0.0%) | 4 (11.8%) | 1 (5.9%) | 0.743^1^ |
|  | Death during hospitalization, n (%) | 2 (66.7%) | 1 (2.9%) | 2 (11.8%) | 0.010^1^ |
|  | Hospital LOS days |  |  |  | 0.018^2^ |
|  | N | 3 | 34 | 17 |  |
|  | Median | 3.0 | 8.5 | 14.0 |  |
|  | Range | 1.0, 3.0 | 1.0, 113.0 | 4.0, 72.0 |  |
|  | ICU LOS days |  |  |  | 0.191^2^ |
|  | N | 3 | 34 | 17 |  |
|  | Median | 0.0 | 2.5 | 4.0 |  |
|  | Range | 0.0, 1.0 | 0.0, 59.0 | 0.0, 46.0 |  |
|  | Total admission cost ($) |  |  |  | 0.736^2^ |
|  | N | 3 | 32 | 16 |  |
|  | Median | 39038.4 | 34805.4 | 33138.7 |  |
|  | Range | 14282.5, 79550.6 | 10011.9, 120969.3 | 1005.0, 168643.1 |  |
|  | PRBC transfusion (mL) |  |  |  | 0.286^2^ |
|  | N |  | 6 | 4 |  |
|  | Median |  | 1582.0 | 806.5 |  |
|  | Range |  | 500.0, 7700.0 | 350.0, 4550.0 |  |
| **Therapeutic embolization** |  | **(N=8)** | **(N=15)** | **(N=4)** |  |
|  | 30-day rebleeding, n (%) | 1 (12.5%) | 8 (57.1%) | 2 (50.0%) | 0.120^1^ |
|  | Death within 30-day of discharge, n (%) | 0 (0.0%) | 1 (6.7%) | 0 (0.0%) | 1.000^1^ |
|  | Death during hospitalization, n (%) | 2 (25.0%) | 3 (20.0%) | 0 (0.0%) | 0.820^1^ |
|  | Hospital LOS days |  |  |  | 0.082^2^ |
|  | N | 8 | 15 | 4 |  |
|  | Median | 6.0 | 15.0 | 10.5 |  |
|  | Range | 1.0, 15.0 | 4.0, 40.0 | 10.0, 55.0 |  |
|  | ICU LOS days |  |  |  | 0.454^2^ |
|  | N | 8 | 15 | 4 |  |
|  | Median | 3.0 | 5.0 | 3.5 |  |
|  | Range | 1.0, 15.0 | 0.0, 37.0 | 3.0, 15.0 |  |
|  | Total admission cost ($) |  |  |  | 0.851^2^ |
|  | N | 7 | 15 | 4 |  |
|  | Median | 28264.0 | 37809.4 | 33175.3 |  |
|  | Range | 4281.0, 77196.0 | 1000.0, 97875.0 | 14574.4, 119366.7 |  |
|  | PRBC transfusion (mL) |  |  |  | 0.755^2^ |
|  | N | 3 | 6 | 2 |  |
|  | Median | 1050.0 | 2209.0 | 1319.0 |  |
|  | Range | 700.0, 1050.0 | 286.0, 3150.0 | 563.0, 2075.0 |  |
| ^1^Fisher Exact p-value; ^2^Kruskal-Wallis p-value | | | | | |
